# Cerebral and pulmonary lymphomatoid granulomatosis and EBV positive oesophageal ulcer in an immunosuppressed renal transplant patient staged and followed with serial MRI and ^18^F-FDG PET/CT after rituximab therapy

**DOI:** 10.1259/bjrcr.20150503

**Published:** 2016-07-28

**Authors:** William Makis, Jean Deschenes

**Affiliations:** ^1^Department of Diagnostic Imaging, Cross Cancer Institute, Edmonton, AB, Canada; ^2^Department of Pathology, Cross Cancer Institute, Edmonton, AB, Canada

## Abstract

Lymphomatoid granulomatosis is a rare Epstein–Barr virus-related lymphoproliferative disorder. We describe a case of a 42-year-old female with lupus nephritis and immunosuppression post renal transplant, who was diagnosed with central nervous system and lung lymphomatoid granulomatosis, as well as an Epstein–Barr virus-positive oesophageal ulcer, and was staged and followed up long term with multiple ^18^F-fludeoxyglucose positron emission tomography/CT scans and brain MRIs after achieving a complete metabolic response with rituximab.

## Case report

**Figure 1.**
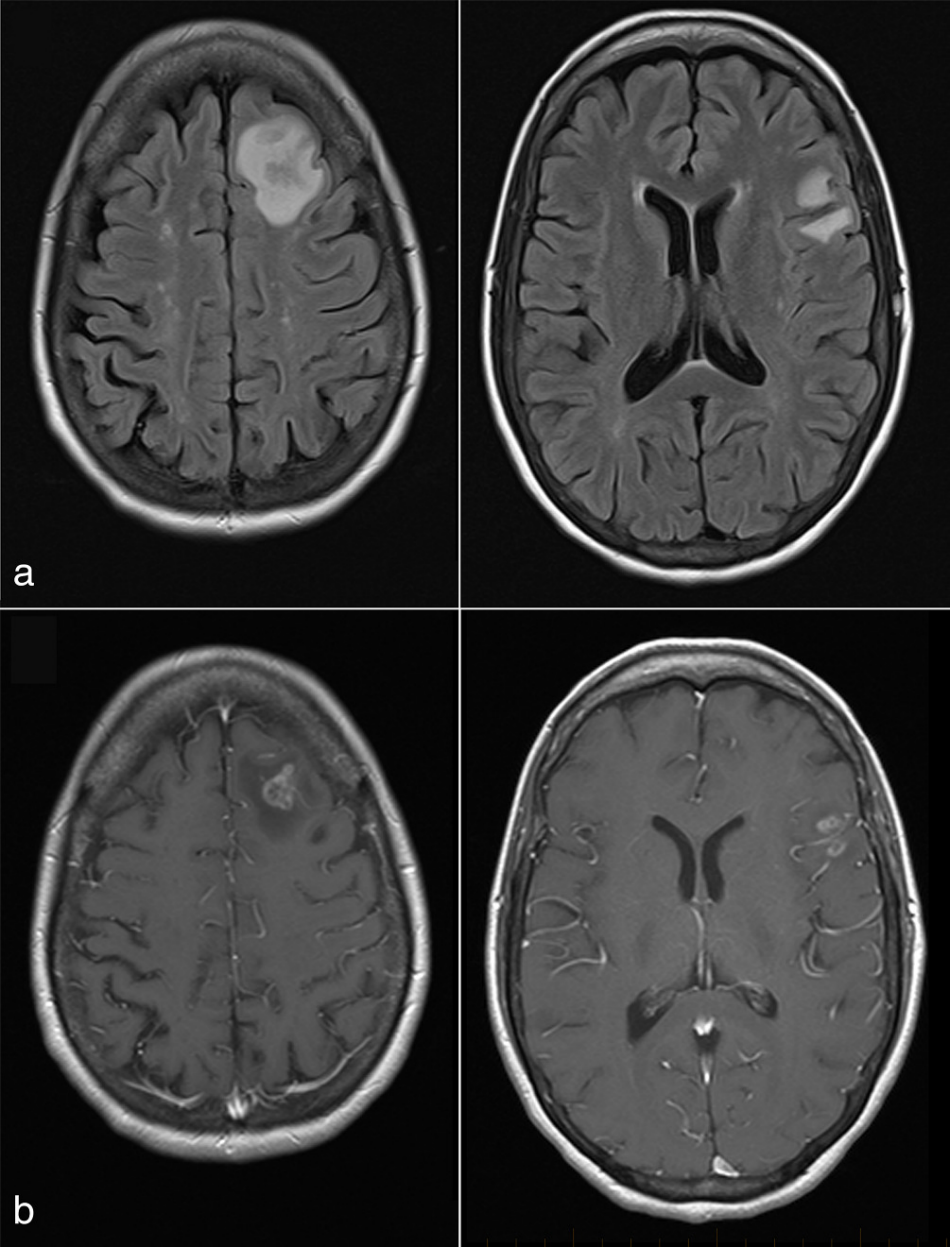
(a) Fluid-attenuated inversion-recovery and (b) *T*_1 _weighted contrast-enhanced MRI showing a 1.4-cm irregular ring-like enhancing lesion within the superior left frontal lobe at the grey–white junction (*T*_1_ and *T*_2_ isointense with surrounding vasogenic oedema) and a 1.3-cm lesion with a similar appearance in the region of the frontal operculum, also at the grey–white interface.

**Figure 2.**
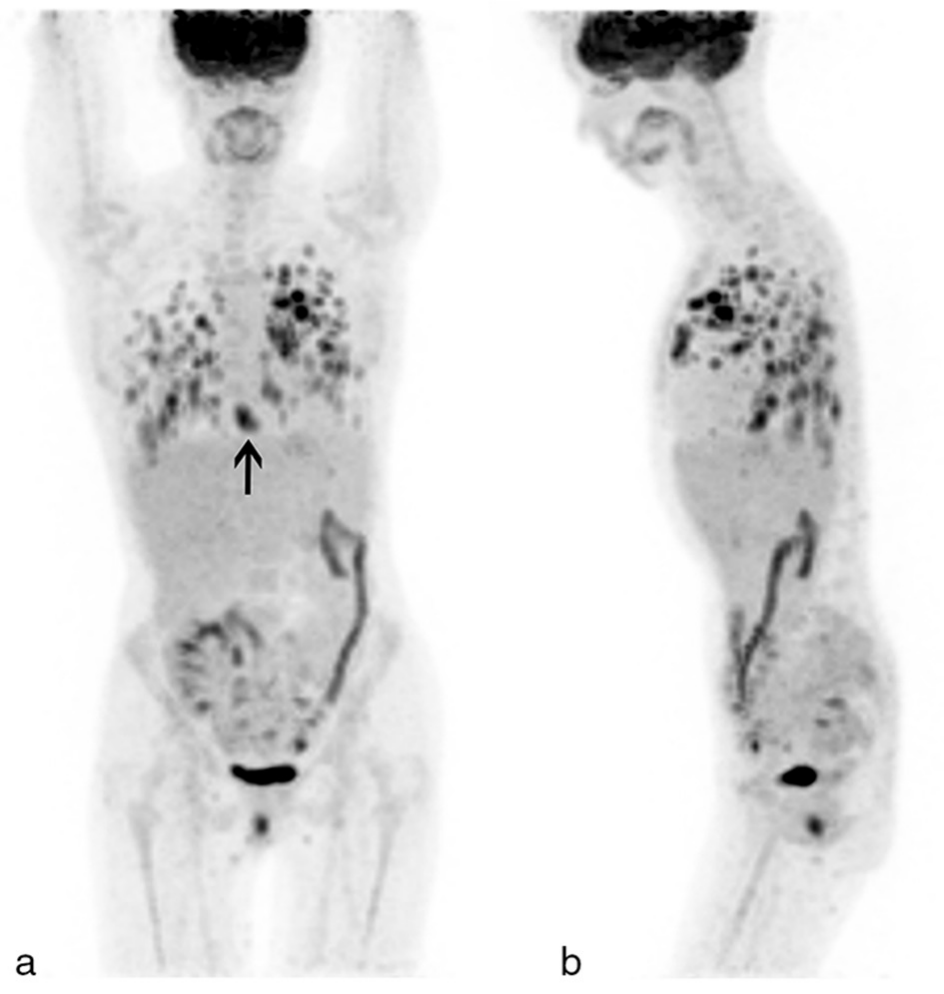
^18^F-fludeoxyglucose positron emission tomography/CT scan (Biograph, Siemens Medical Solutions, Knoxville, TN) maximum intensity projection (a) anterior and (b) left lateral images showing innumerable ^18^F-fludeoxyglucose-avid lung lesions (SUV_max_ 12.1) and a distal oesophageal lesion (SUV_max_ 8.5) [arrow in (a)]. SUVmax, maximum standardized uptake value.

**Figure 3.**
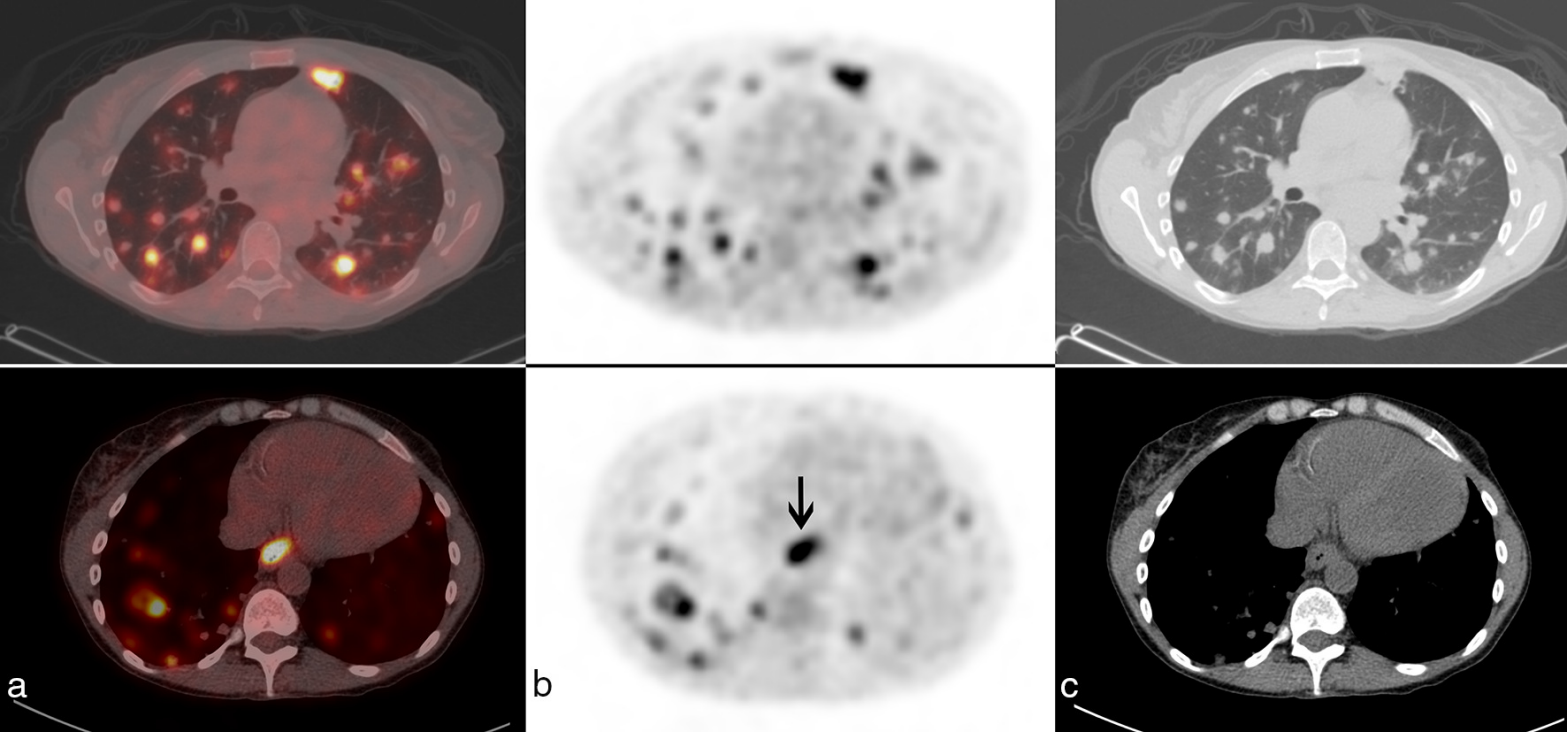
Transaxial (a) positron emission tomography/CT fusion, (b) positron emission tomography and (c) CT images showing the innumerable ^18^F-fludeoxyglucose-avid lung nodules, which were irregular but well defined on CT images, and a distal oesophageal focus of intense ^18^F-fludeoxyglucose uptake [arrow in (b)].

**Figure 4.**
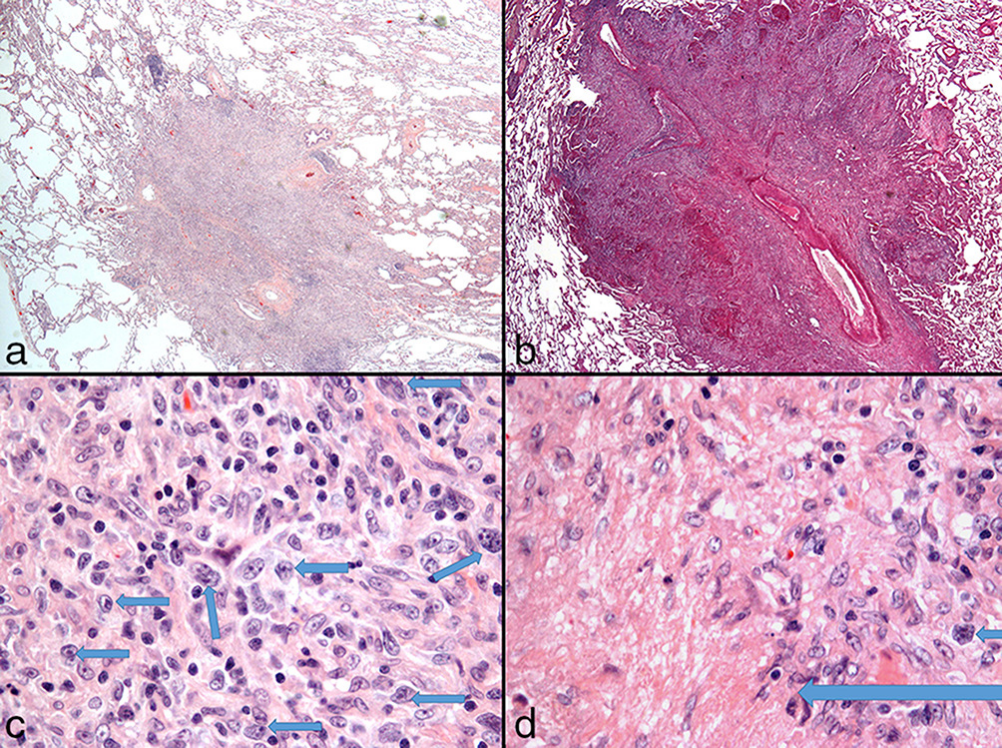
Lung wedge biopsy. (a) Low power (OM 20×) haematoxylin and eosin stain of a lung nodule; (b) low power (OM 20×) periodic acid–Schiff stain showing angiocentric pattern and areas of necrosis and granulomas, consistent with Grade 3 lymphomatoid granulomatosis; (c) high power (OM 500×) showing numerous large atypical cells (arrows); (d) (OM 500×) showing an atypical cell (small arrow) and necrosis with palisaded granulomatous histiocytes (large arrow). OM, original magnification.

**Figure 5.**
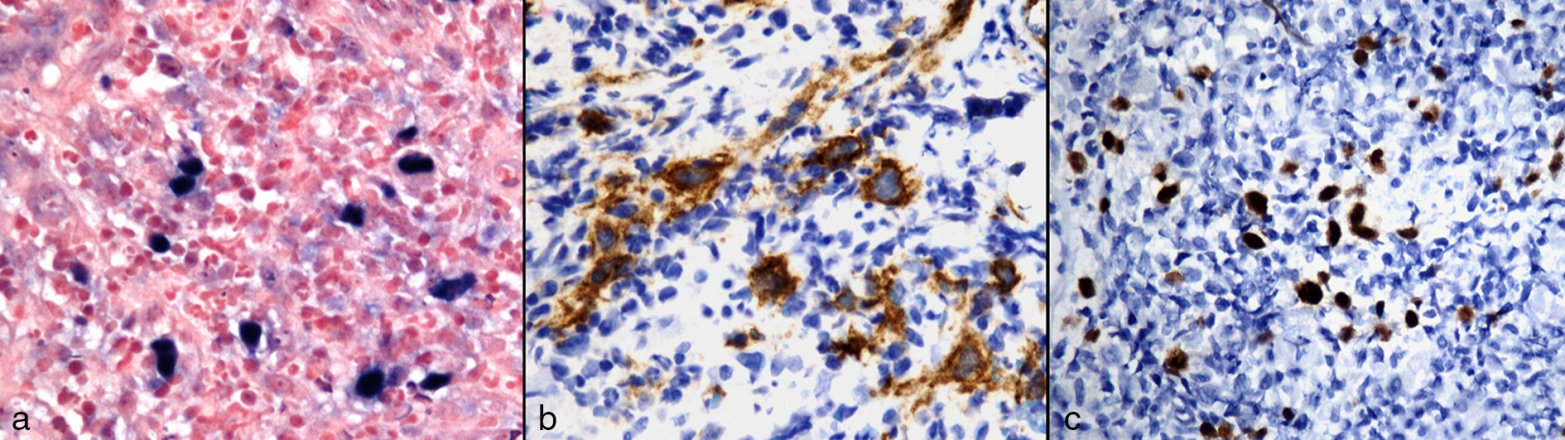
Positive Epstein–Barr virus-encoded RNA staining (OM 500×); (b) positive CD30 staining (OM 500×) and (c) positive PAX5 staining (OM 400×). OM, original magnification; PAX5, paired box 5.

**Figure 6.**
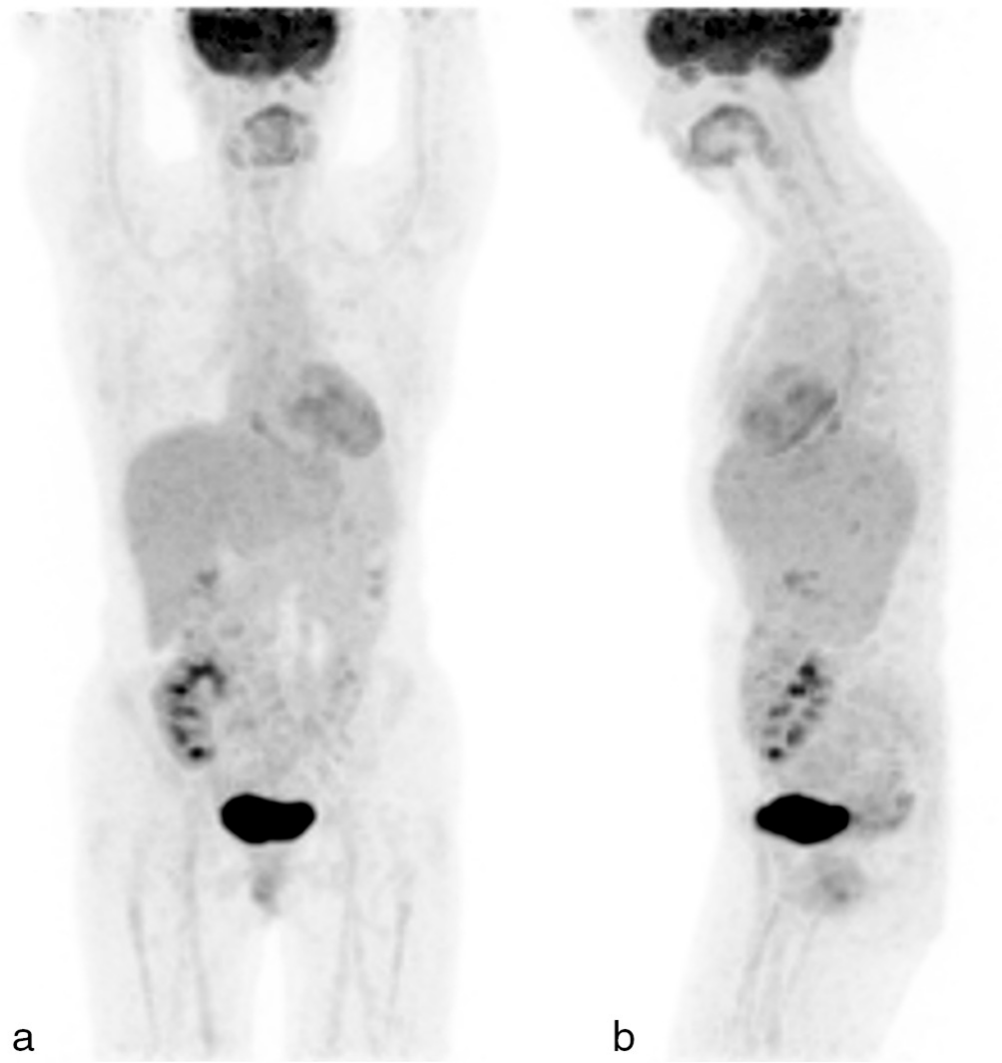
A follow-up ^18^F-fludeoxyglucose positron emission tomography/CT performed 4 weeks after completion of the fourth cycle of rituximab. (a) Anterior and (b) lateral maximum intensity projection images showing complete metabolic resolution of all of the lung nodules as well as the Epstein–Barr virus-positive oesophageal ulcer.

**Figure 7.**
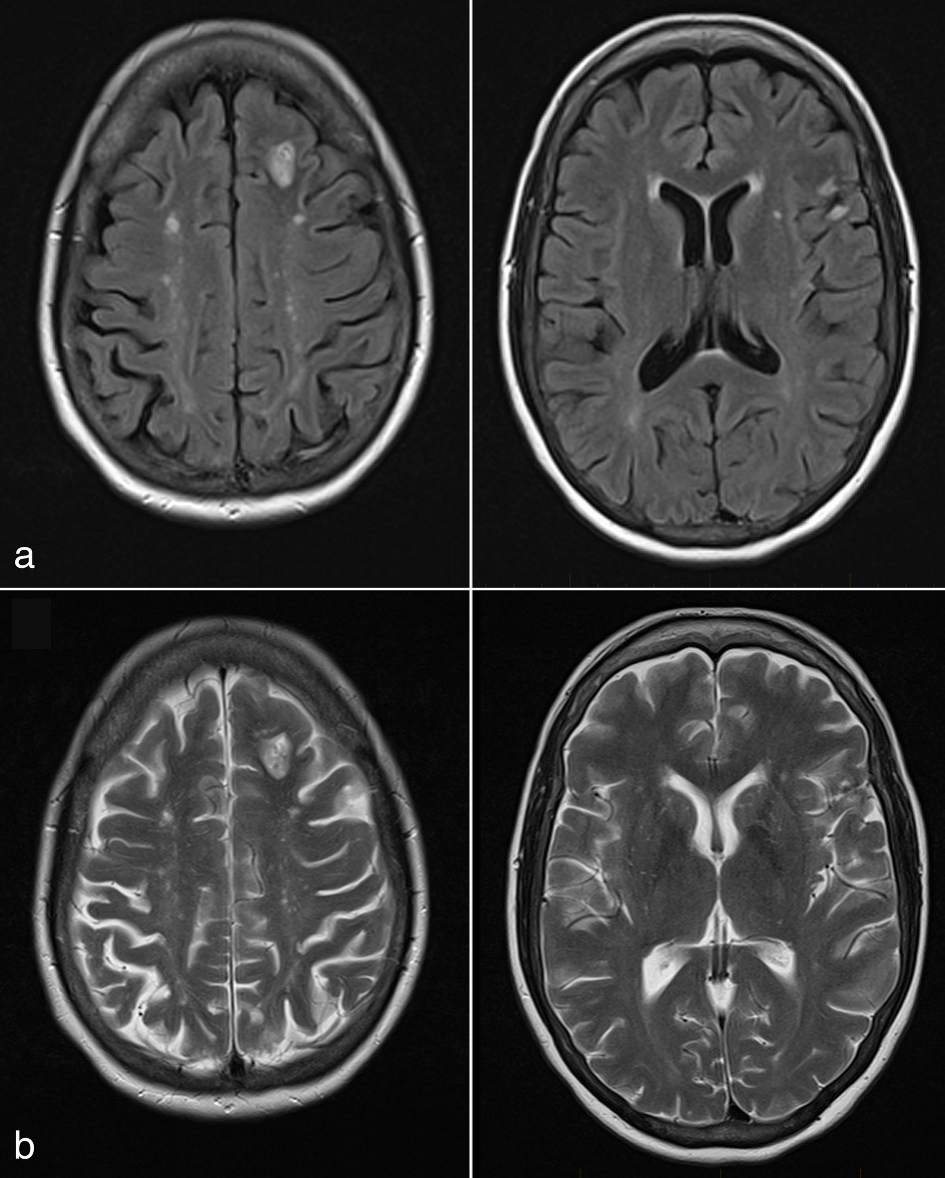
(a) Fluid-attenuated inversion-recovery and (b) *T*_2_ weighted images. A follow-up MRI performed at the same time as follow-up positron emission tomography/CT scan showed that the previously seen left superior frontal and left inferior frontal lesions had resolved.

**Figure 8.**
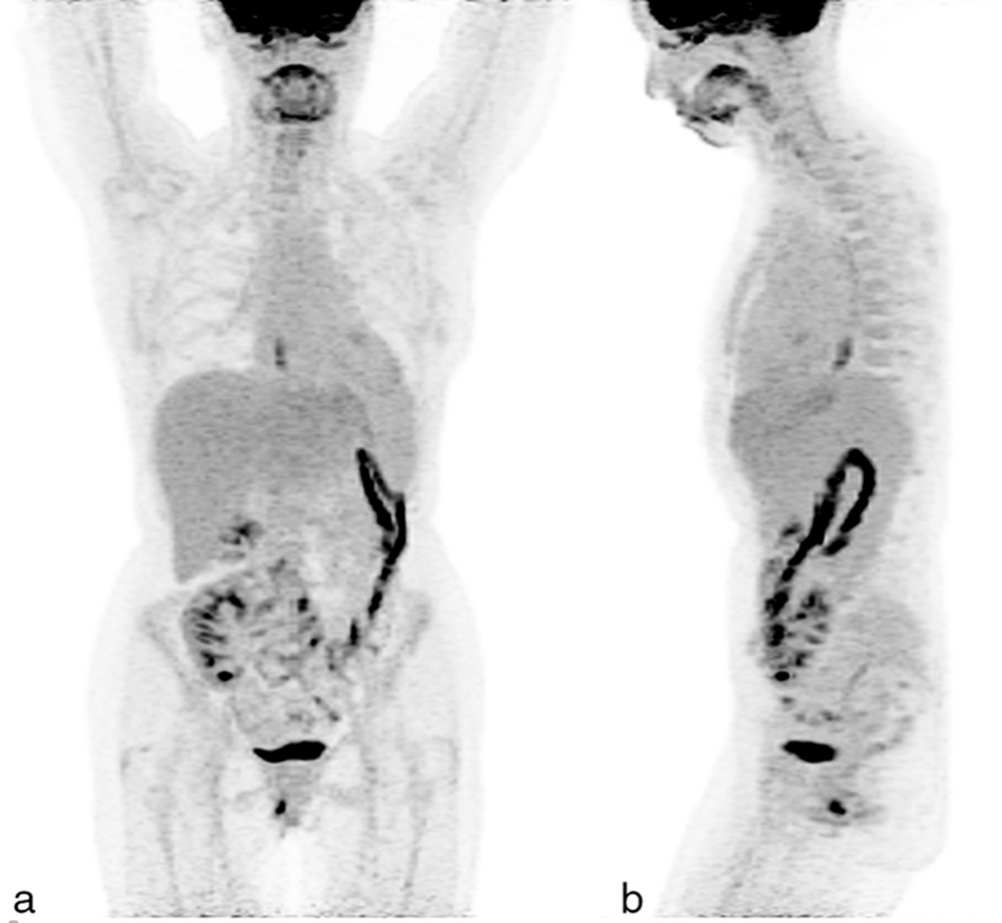
A follow-up ^18^F-fludeoxyglucose positron emission tomography/CT scan performed 12 months after the completion of rituximab therapy. (a) Anterior and (b) lateral maximum intensity projection images do not show any ^18^F-fludeoxyglucose-avid lung lesions, confirming disease remission.

**Figure 9.**
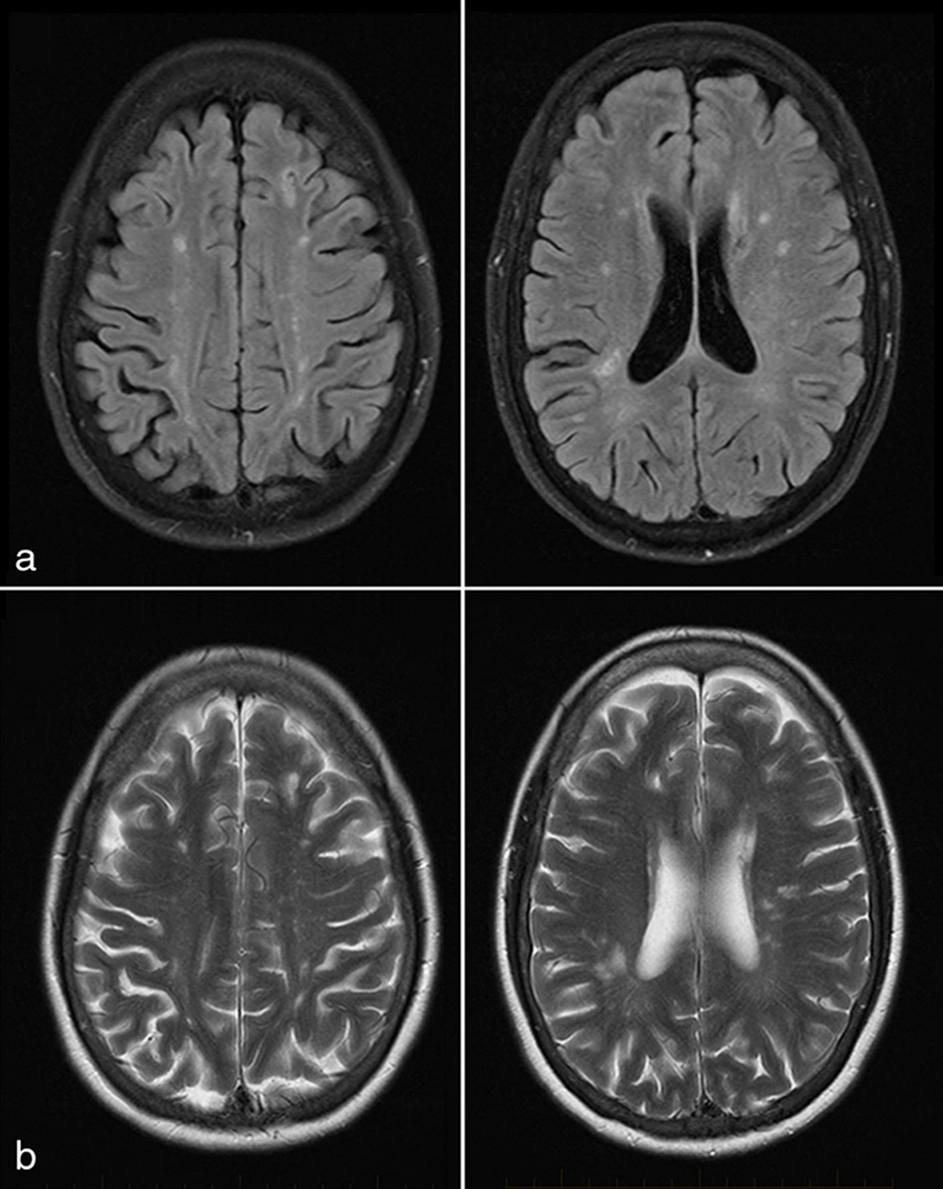
(a) Fluid-attenuated inversion-recovery and (b) *T*_2_ weighted images. A follow-up MRI performed 12 months after completion of rituximab therapy did not show any brain lesions.

A 42-year-old female with a history of systemic lupus erythematosus diagnosed at age 16 years (treated with azathioprine, cyclophosphamide and prednisone) and a renal transplant owing to lupus nephritis at age 30 years (treated with mycophenolate mofetil) presented with sudden onset of confusion and difficulty finding words. Contrast-enhanced MRI of the brain revealed two enhancing brain lesions (Figure 1), and the patient was referred for ^18^F-fludeoxyglucose positron emission tomography/CT (^18^F-FDG PET/CT) imaging, which showed innumerable ^18^F-FDG-avid lung lesions with a maximum standardized uptake value of 12.1 (Figures 2 and 3), which were confirmed to be Grade 3/3 lymphomatoid granulomatosis (LYG) on lung wedge biopsy (Figure 4) [certain nodules showed >100 Epstein–Barr virus (EBV)-encoded RNA-positive cells per high power field, Figure 5]. There was also intense focal ^18^F-FDG uptake in the distal oesophagus with a maximum standardized uptake value of 8.5, which prompted a gastroscopy and biopsy, revealing an EBV-positive oesophageal ulcer that was treated with long-term valganciclovir 450 mg by mouth daily. She was treated with 4 weekly cycles of rituximab. A follow-up PET/CT scan performed 4 weeks after completion of rituximab showed complete metabolic resolution of LYG lung lesions as well as the EBV oesophageal ulcer (Figure 6), and a follow-up MRI revealed complete resolution of the brain lesions (Figure 7). Surveillance PET/CT (Figure 8) and MRI (Figure 9) studies performed 12 months later confirmed disease remission.

## Discussion

LYG is an EBV-related lymphoproliferative disorder characterized by an angiocentric and angiodestructive infiltration of the lungs, nervous system and skin with a polymorphous infiltrate of lymphocytes, plasma cells, histiocytes and atypical lymphoreticular cells. While the pathogenesis of LYG remains unknown, some investigators consider LYG as a neoplastic B-cell process correlated to EBV antigen stimulation, as EBV has been found in most cases of LYG.^1^ At onset, most patients are between the ages of 30–50 years, with a predominance of males to females of 6–7 to 1.^2^ Patients are often immunocompromised. LYG predominantly involves the lungs, and extrapulmonary involvement is seen in the skin (39%) and the central nervous system (CNS; 30%), and can be associated with splenomegaly, hepatomegaly or lymphadenopathy.^3^ Patients can present with cough, shortness of breath, chest pain and B symptoms (fever, weight loss, malaise).^4^

^18^F-FDG PET/CT findings vary considerably. Reports in the literature describe a single ^18^F-FDG-avid nodule,^5^ few mildly ^18^F-FDG-avid nodules,^6^ a large cavitating ^18^F-FDG-avid mass,^7,8^ multiple cavitating masses^9^ and numerous fluffy nodules.^10^ Staging, evaluation of response to therapy and long-term follow-up of LYG patients with ^18^F-FDG PET/CT has not been previously described in the literature and warrants further investigation. Brain lesions do not show increased ^18^F-FDG uptake and are best evaluated with MRI.^11,12^

Lung nodules are present in up to 80% of cases. In most cases, the lung nodules are irregular but well defined. They tend to be distributed along the bronchovascular bundles or interlobular septa, which can be explained by the angiocentric distribution of LYG. The differential diagnosis of the CT scan findings in the lungs includes pseudolymphoma, malignant lymphoma, lymphocytic interstitial pneumonia, metastasis, sarcoidosis, Wegener’s granulomatosis and cryptogenic organizing pneumonia.^13^ The most common CNS findings of LYG on MRI scans are multiple focal intraparenchymal lesions with high signal intensity on *T*_2_ weighted images and punctate and/or linear enhancement, which seems to be a characteristic finding of LYG in the brain and corresponds to known perivascular and vascular wall infiltration by lymphoid cells.^14^

EBV is most prevalent in Grade 3/3 lesions compared with Grade 1 or 2, and although the natural course of Grade 3 disease is extremely variable, prognosis is poor and mortality is very high. Katzenstein et al^3^ found that 63.5% of 152 patients with LYG died within 36 months owing to destruction of pulmonary parenchyma, sepsis, massive haemoptysis or CNS disorders. Approximately 12% of LYG patients also develop malignant lymphoma.^3^ Experimental treatment options for LYG have included corticosteroids, antiviral therapy, interferon-α and chemotherapy. The best results described in the literature have been reported with rituximab, an anti-CD20 monoclonal antibody. The rationale for the use of rituximab is the expression of CD20 on lymphoid cells of LYG lesions and the well-documented efficacy of rituximab in other CD20-positive lymphomas.^15^ Several studies have now shown that CNS LYG lesions also respond to rituximab, as approximately 1% of administered rituximab can pass through the blood–brain barrier.^16^

## Learning points

1. ^18^F-FDG PET/CT scan is useful in staging LYG in combination with brain MRI if a patient also presents with neurological symptoms.
2. ^18^F-FDG PET/CT scan can detect EBV-positive oesophageal ulcers and can direct their management.
3. Treatment response of LYG to rituximab therapy can be evaluated with ^18^F-FDG PET/CT scan.
4. ^18^F-FDG PET/CT scan is useful in the long-term follow-up of LYG patients who have an extremely variable disease course, poor prognosis and an elevated risk of developing lymphoma.

## Consent

Informed consent to publish this case (including images and data) was obtained and is held on record.
